# Automatic meter classification of Kurdish poems

**DOI:** 10.1371/journal.pone.0280263

**Published:** 2023-02-01

**Authors:** Aso Mahmudi, Hadi Veisi

**Affiliations:** Faculty of New Sciences and Technologies, University of Tehran, Tehran, Iran; University of Kurdistan Hewler, IRAQ

## Abstract

Most of the classic texts in Kurdish literature are poems. Knowing the meter of the poems is helpful for correct reading, a better understanding of the meaning, and avoiding ambiguity. This paper presents a rule-based method for the automatic classification of the poem meter for the Central Kurdish language also known as Sorani. The metrical system of Kurdish poetry is divided into three classes quantitative, syllabic, and free verses. As the vowel length is not phonemic in the language, there are uncertainties in syllable weight and meter identification. The proposed method generates all the possible situations and then, by considering all lines of the input poem and the common meter patterns of Kurdish poetry, identifies the most probable meter type and pattern of the input poem. Evaluation of the method on a dataset from VejinBooks Kurdish corpus resulted in 97.3% of precision in meter type and 96.2% of precision in pattern identification.

## 1 Introduction

Kurdish is an Indo-Iranian language spoken by millions of people in western Asia. Among various dialects of Kurdish, in this paper, we focus on the standard literary form of the central dialect group also known as Sorani (Standard Central Kurdish, SCK), which has produced more literary texts than other dialects in the past century [1,2]. A greater part of classical Kurdish literature is in poetry form [1, Ch. 4.1], [3, p. 639], maybe because poems are easier to memorize when powerful neighboring languages (Arabic, Persian and Turkish) confined Kurdish writing in the past centuries.

Identifying the form (rhyming scheme) and meter (rhythmic structure) of poetry is a time-consuming and complicated task for beginners. An automatic application can help students and inexpert poets to learn and correct their mistakes. There are three kinds of Kurdish poem meter: Quantitative (syllable weight rhythm), Syllabic (syllable count rhythm), and Free verses. This paper introduces a rule-based method for the automatic classification of Kurdish poem meters. Since Kurdish lacks large language processing resources, rule-based methods can be leveraged in future data-driven solutions. The method, first, decomposes each line of the poem into its syllables. Then, it identifies the repetition pattern in syllable weight and number. However, poetry-related tasks significantly challenge natural language processing more than any other genre [4]. In this task, there are issues too. In the writing system of Kurdish, correspondence between some graphemes and phonemes is not one-to-one; therefore, the syllabification process confronts ambiguities. In addition, syllable weight is not a distinctive concept in Kurdish, and it is probable to change the weight of some syllables in a poem without altering the meaning.

The proposed method utilizes a rule-based method of SCK grapheme-to-phonemes converter [5], which syllabifies the input poem. Then, for the analysis and detection of the poem meter, we consider all possible patterns of each line. Eventually, we analyze the whole poem to calculate a score for each common quantitative pattern. If a pattern repeats in all lines, the proposed method classifies it as quantitative. Else, if most of the lines have equal syllable count, the poem is a syllabic verse; otherwise, it is a free verse.

The rest of the paper is organized as follows: Section ‎2 reviews phonology and the alphabet of Standard Central Kurdish and the common types of meters of Kurdish poems. Section ‎3 presents the steps of the proposed method for the classification of Kurdish poems. Section ‎4 describes the test dataset and results. Section ‎5 gives conclusions and further works.

## 2 Background and related works

### 2.1 Phonemes, alphabet, and syllables of SCK

There are 37 phonemes in SCK, including 8 vowels and 29 consonants [5]. This study uses the Hawar alphabet (standard Latin script for Northern Kurdish) with changes in some consonants. Table 1 compares IPA and the Standard Arabic alphabet of Kurdish with this study’s transcription of consonants.

**Table 1:** Consonants of Standard Central Kurdish.

*Kurdish Alphabet*	ئ	ب	پ	ت	ج	چ	ح	خ	د	ر	ڕ	ز	ژ	س	ش	ع	غ	ف	ڤ	ق	ک	گ	ل	ڵ	م	ن	و	هـ	ی
*IPA*	ʔ	b	p	t	dʒ	tʃ	ħ	x	d	ɾ	r	z	ʒ	s	ʃ	ʕ	ɣ	f	v	q	k	g	l	ɫ	m	n	w	h	j
*This Study*	ʔ	b	p	t	c	ç	ḧ	x	d	r	ř	z	j	s	ş	ƹ	ẍ	f	v	q	k	g	l	ł	m	n	w	h	y

As the syllable weight is the essential material in the identification of poem meter, we will discuss the SCK vowel’s length more precisely. Table 2 describes the details of Standard Central Kurdish vowels. The long vowels *(/î*, *ê*, *a*, *o*, *û/*) are shorter in final unstressed positions, and the short vowel */e/* in word-final positions can be pronounced longer [6]. The vowel /i/ (bizroke) is unstable in most environments [7] and does not have a grapheme in the standard Kurdish alphabet [5,8].

**Table 2:** Vowels of Standard Central Kurdish.

IPA	This study	Kurdish alphabet	Description	Normal Length
i	î	ی	close front unrounded	long
ɛ	ê	ێ	mid-open front unrounded	long
ä	a	ا	open front-central unrounded	long
o̞	o	ۆ	mid back rounded	long
u	û	وو	close back rounded	long
ʊ	u	و	half-close back-central rounded	short
a	e	ە	open front unrounded	short
ɪ	i		half-close front-central unrounded	short

The Kurdish alphabet, which is adapted from the Perso-Arabic script, has ambiguities in three cases [5]:

- The letter “ی” indicates both consonant */y/* and vowel */î/*.
- The letter “و” can represent the consonant */w/* and the short vowel */u/*. In addition, if this letter comes up twice (“وو”), it can be long vowel */û/* or combinations */uw/*, */uw/*, or */ww/*.
- There is no letter for the short vowel */i/* (bizroke) in the Arabic script of Kurdish.

In the syllable structure of Kurdish, the nucleus is always a vowel, and the onset is one or two consonants. In two-consonant onsets, the second consonant must be */w/* or */y/*. Coda has zero to three consonants. Three-consonant coda is rare, occurs only in some dialects [9], and was not observed in our dataset of Kurdish poetry. Table 3 presents syllable types and their normal weight in SCK.

**Table 3:** Syllable Types in Standard Central Kurdish.

No.	Syllable Type	Example	Normal Weight
1	CV	*/be/* (‘with’) */ba/* (‘wind’)	light heavy
2	CVC	*/ber/* (‘front’)	heavy
3	CVCC	*/berd/* (‘stone’)	heavy
4	CVcCC	*/řoyşt/* (‘went’)	heavy
5	CcV	*/xwê/* (‘salt’)	heavy
6	CcVC	*/xwên/* (‘blood’)	heavy
7	CcVCC	*/xwênd/* (‘read’)	heavy

Note: c = approximant, C = other consonants, V = Vowel.

### 2.2 Types of Kurdish poems

As mentioned, the Kurdish language is a collection of dialects whose speakers live in Iran, Iraq, Turkey, Syria, and parts of the Caucasus. Neighboring different nations has led Kurdish literature to enjoy the characteristics of different literature styles.

In classical literature of Kurdish, there are three categories of poetic works: “quantitative (Arudi) verse”, “beit (syllabic songs)” and “gorani (lyric songs)” [10]. Kurdish quantitative verses are an imitation of Arabic and especially Persian poetry [11], and in terms of meter, it is based on syllable weight, i.e., all lines of a poem have an equal number of syllables, repeating a pattern of light and heavy syllables [12]. Beit and gorani have “syllabic meter”. The syllabic or numerical meter is rooted in the ancient tradition of Iranian languages, and it has long existed among different ethnic groups in Iran [10]. There is evidence of a syllabic meter in pre-Islamic literature in the texts of the Zoroastrian and Manichaean rituals [10,13,14]. In a syllabic meter, the weight of the syllables and the place of stress do not affect the meter, and only the total number of syllables in each line is important.

Fixed-form poems in Kurdish consist of lines that have an equal number of syllables. In most of the forms, like ghazal and mathnawi, even lines rhyme; however, in some forms, like mukhammas, rhyming is different. In the modern literature of Kurdish, “free verse” is a new style that is not limited to a fixed form, and the number of syllables in each line may be different [15]. This study considers three types of Kurdish poems: Quantitative, Syllabic, and Free verses.

#### 2.2.1 Syllabic verses

In syllabic or numerical verses, only the number of syllables in feet is considered, and the syllable weight sequence is not following a specific pattern. Kurdish folk poems are syllabic verses [16]. There are three types of three, four, and five-syllable feet in Kurdish syllabic verses, which are repeated uniformly or alternately at each line [12]. Table 4 shows the types of syllabic verses in Kurdish and how feet are combined to form each line. The most common type of syllabic verses in Kurdish is 10-syllabic [10, p. 15], [12, p. 247].

**Table 4:** Types of syllabic verses in Kurdish poetry (Frequencies from VejinBooks corpus, up to 2019/12/1).

Type	Feet Order	Frequency
5-syllabic	= 5	0
6-syllabic	= 3+3	0
7-syllabic	= 4+3	34
8-syllabic	= 4+4	159
9-syllabic	= 3+3+3	0
10-syllabic	= 5+5	2,020
11-syllabic	= 4+4+3	60
12-syllabic	= 4+4+4 or = 3+3+3+3	14
13-syllabic	= 4+4+5	23
14-syllabic	= 4+3+4+3	31
15-syllabic	= 5+5+5 or = 4+4+4+3	17
16-syllabic	= 4+4+4+4	19

#### 2.2.2 Quantitative verses

The quantitative meter is an arrangement of heavy (ˉ) and light (˘) syllables in a line of the poem, as is found in Greek and Latin poems [17]. This type of meter fits languages like Arabic, where the vowel length is distinctive and changes the meaning. Arabic has three short vowels [a i u] with distinctive long pairs [aː iː uː] [18]. For example, in the following Arabic hemistich by Hafez (1325–1390), all vowels are pronounced with their normal lengths:

« اَلا يا اَيُّهَا السّاقي اَدِرْ کَأسَاً و ناوِلْها »

**Table:** 

syllables:	*ʔa*	*laː*	*yaː*	*ʔay*	*yu*	*has*	*saː*	*qiː*	*ʔa*	*dir*	*kaʔ*	*san*	*wa*	*naː*	*wil*	*haː*
	˘	ˉ	ˉ	ˉ	˘	ˉ	ˉ	ˉ	˘	ˉ	ˉ	ˉ	˘	ˉ	ˉ	ˉ

However, in languages such as Persian and Kurdish, whose vowel length is not distinctive, to follow the metrical pattern of the poem, some syllables can be pronounced contrary to their natural weight [19]. For example, in another hemistich of that poem which is in Persian, the short vowel */e/* in the word */ha*.*me/* ‘all’ should be pronounced as */ha*.*meː/* to preserve the meter:

«همه کارم ز خودکامی به بدنامی کشید آخر»

**Table:** 

syllables:	*hæ*	*meː*	*kɒː*	*ræm*	*ze*	*xod*	*kɒː*	*miː*	*be*	*bæd*	*nɒː*	*miː*	*ke*	*šiː*	*dɒː*	*xer*
	˘	ˉ	ˉ	ˉ	˘	ˉ	ˉ	ˉ	˘	ˉ	ˉ	ˉ	˘	ˉ	ˉ	ˉ

In Kurdish, as shown in Table 3, only syllable with a single consonant onset and short vowel nucleus and no coda (e.g., */be/*) are light, and all other types of syllables are heavy. In Kurdish quantitative verses, such as the following line by Qani’ (1898–1965), syllable weights are regularly pronounced as their natural weights:

«لەباتی من بڵێن بولبول نەخوێنێ قەت بە مل گوڵدا»

**Table:** 

syllables:	*le*	*ba*	*tî*	*min*	*bi*	*łên*	*bul*	*bul*	*ne*	*xwê*	*nê*	*qet*	*be*	*mil*	*guł*	*da*
	˘	ˉ	ˉ	ˉ	˘	ˉ	ˉ	ˉ	˘	ˉ	ˉ	ˉ	˘	ˉ	ˉ	ˉ

However, to save the meter, some syllables (4, 5, and 11) are pronounced differently, as in the following line by Piramerd (1867–1950) from the meter “**ˉˉ˘/ˉ˘ˉ˘/˘ˉˉ˘/ˉ˘ˉ**”:

«چەند ساڵ گوڵی ھیوای ئێمە پێ پەست بوو تاکو پار»

**Table:** 

	_1_	_2_	_3_	_4_	_5_	_6_	_7_	_8_	_9_	_10_	_11_	_12_	_13_	_14_
syllables:	*çend*	*sał*	*gu*	*łî*	*hî*	*way*	*ʔê*	*me*	*pê*	*pest*	*bû*	*ta*	*ku*	*par*
normal weights:	ˉ	ˉ	˘	˘	ˉ	ˉ	ˉ	˘	ˉ	ˉ	ˉ	ˉ	˘	ˉ
meter pattern:	ˉ	ˉ	˘	ˉ	˘	ˉ	˘	˘	ˉ	ˉ	˘	ˉ	˘	ˉ

Table 5 shows the most common patterns of Kurdish quantitative verses extracted from the VejinBooks corpus [20].

**Table 5:** Common patterns of Kurdish quantitative verses (VejinBooks corpus, up to 2019/12/1).

Rank	Pattern Title	Syllable Weight Pattern	Freq.	%
*1*	فاعلاتن فاعلاتن فاعلاتن فاعلن	ˉ˘ˉˉ/ˉ˘ˉˉ/ˉ˘ˉˉ/ˉ˘ˉ	1044	27.14
*2*	مفاعیلن مفاعیلن مفاعیلن مفاعیلن	˘ˉˉˉ/˘ˉˉˉ/˘ˉˉˉ/˘ˉˉˉ	999	25.97
*3*	مفاعیلن مفاعیلن فعولن	˘ˉˉˉ/˘ˉˉˉ/˘ˉˉ	386	10.03
*4*	مفعولُ مفاعیلُ مفاعیلُ فعولن	ˉˉ˘/˘ˉˉ˘/˘ˉˉ˘/˘ˉˉ	334	8.68
*5*	مفعولُ مفاعیلُ مفاعیلُ فعل	ˉˉ˘/˘ˉˉ˘/˘ˉˉ˘/˘ˉ	272	7.07
*6*	مفعولُ فاعلاتُ مفاعیلُ فاعلن	ˉˉ˘/ˉ˘ˉ˘/˘ˉˉ˘/ˉ˘ˉ	213	5.54
*7*	فعلاتن فعلاتن فعلاتن فعلن	˘˘ˉˉ/˘˘ˉˉ/˘˘ˉˉ/˘˘ˉ	138	3.59
*8*	مفعولُ مفاعلن فعولن	ˉˉ˘/˘ˉ˘ˉ/˘ˉˉ	131	3.41
*9*	فاعلاتن فاعلاتن فاعلن	ˉ˘ˉˉ/ˉ˘ˉˉ/ˉ˘ˉ	62	1.61
*10*	فعلاتن مفاعلن فعلن	˘˘ˉˉ/˘ˉ˘ˉ/˘˘ˉ	45	1.17
*11*	مفاعلن فعلاتن مفاعلن فعلن	˘ˉ˘ˉ/˘˘ˉˉ/˘ˉ˘ˉ/˘˘ˉ	40	1.04
*12*	مفعولُ مفاعیلن مفعولُ مفاعیلن	ˉˉ˘/˘ˉˉˉ/ˉˉ˘/˘ˉˉˉ	31	0.81
*13*	مفعولُ فاعلاتن مفعولُ فاعلاتن	ˉˉ˘/ˉ˘ˉˉ/ˉˉ˘/ˉ˘ˉˉ	28	0.73
*14*	فعلاتن فعلاتن فعلن	˘˘ˉˉ/˘˘ˉˉ/˘˘ˉ	20	0.52
*15*	مستفعلن مستفعلن مستفعلن مستفعلن	ˉˉ˘ˉ/ˉˉ˘ˉ/ˉˉ˘ˉ/ˉˉ˘ˉ	19	0.49
*16*	فعولن فعولن فعولن فعل	˘ˉˉ/˘ˉˉ/˘ˉˉ/˘ˉ	14	0.36
*17*	مفاعلن فعولن مفاعلن فعولن	˘ˉ˘ˉ/˘ˉˉ/˘ˉ˘ˉ/˘ˉˉ	13	0.34
*18*	مفتعلن فاعلن مفتعلن فاعلن	ˉ˘˘ˉ/ˉ˘ˉ/ˉ˘˘ˉ/ˉ˘ˉ	9	0.23
*19*	مفتعلن مفتعلن فاعلن	ˉ˘˘ˉ/ˉ˘˘ˉ/ˉ˘ˉ	8	0.21
*20*	فعولن فعولن فعولن فعولن	˘ˉˉ/˘ˉˉ/˘ˉˉ/˘ˉˉ	8	0.21
*21*	فاعلاتن فاعلاتن فاعلاتن فاعلاتن	ˉ˘ˉˉ/ˉ˘ˉˉ/ˉ˘ˉˉ/ˉ˘ˉˉ	7	0.18
*22*	مفتعلن مفاعلن مفتعلن مفاعلن	ˉ˘˘ˉ/˘ˉ˘ˉ/ˉ˘˘ˉ/˘ˉ˘ˉ	7	0.18
*23*	مفاعلن مفاعلن مفاعلن مفاعلن	˘ˉ˘ˉ/˘ˉ˘ˉ/˘ˉ˘ˉ/˘ˉ˘ˉ	7	0.18
*24*	مفاعیلُ مفاعیلُ مفاعیلُ فعولن	˘ˉˉ˘/˘ˉˉ˘/˘ˉˉ˘/˘ˉˉ	5	0.13
*25*	متفاعلن متفاعلن متفاعلن متفاعلن	˘˘ˉ˘ˉ/˘˘ˉ˘ˉ/˘˘ˉ˘ˉ/˘˘ˉ˘ˉ	3	0.08
*26*	مفاعلن فعلاتن مفاعلن فعلاتن	˘ˉ˘ˉ/˘˘ˉˉ/˘ˉ˘ˉ/˘˘ˉˉ	2	0.05
*27*	فعلاتُ فاعلاتن فعلاتُ فاعلاتن	˘˘ˉ˘/ˉ˘ˉˉ/˘˘ˉ˘/ˉ˘ˉˉ	2	0.05

Aziz Gardi [15] has also conducted a comprehensive statistical study on quantitative verses of 82 Kurdish poets. Future works will benefit from its information.

### 2.3 Meter classification in Kurdish poetry

The principles of classification of quantitative meter in Kurdish are similar to Persian [12]. Experts of Persian poetry use the following traditional steps for the identification of meter in quantitative verses [19,21]:

1. **Scansion**: each line will be divided into its syllables.
2. **Comparing**: light and heavy syllables sequence is compared with the known common meter patterns.
3. **Considering poetic license**: Sometimes, it is necessary to make changes in the pronunciation of certain words in order to match the overall meter of the poem, such as making a light syllable heavy, lightening a heavy syllable, and fading together two adjacent words.

If a pattern is repeated in all lines, the poem will be recognized as a quantitative verse, and that pattern is proposed as the poem’s meter. Otherwise, if all lines have an equal number of syllables, then the poem will be recognized as a syllabic verse of that number. Else, when lines have neither a consistent pattern nor an equal number of syllables, then the poem is free verse [12].

### 2.4 Related works

As far as the authors know, no research has been done on the automatic classification of Kurdish poetry. Considering the similarities between the Kurdish quantitative verses and classic Persian, Arabic, and Turkish ones, we will give a brief overview of the works done in these languages.

In the Arabic and Persian orthographies, short vowels are written only for kids or ritual texts. The absence of short vowels in poems is a challenge for the syllabification step [22]. Mojiri [23], Kurt & Kara [24], Alabbas et al. [25], and Abuata & Al-Omari [26] have considered preprocessing steps for insertion of short vowels (diacritizing) and turning the text into phonemic representation. For example, The Basrah system [25] converts the word like “سدّ” to “سَدْد” and “والشمس” into “وَشْشمس”. Mojiri [23] looks up the words that cannot be syllabified by the rules from a transliteration dictionary. Jafari Qamsari [27] relies on the distributive characteristics of Persian phonemes and by using poetic and phonetic rules, converts the input Persian couplet into light, heavy, and potentially heavy syllable string.

Recently, data-driven and machine-learning works have been done on Arabic and Persian meter classification. Yousef et al. [28] encode the input poem at the character level and directly fed it to the recurrent neural networks without feature handcrafting. Yousefi [29] finds the unwritten linking vowel (izafe) by convolutional neural networks. Al-shaibani et al. [30] by deep bidirectional recurrent neural networks classify the meter of Arabic poems without diacritizing. Abandah et al. [31] use recurrent neural networks with bidirectional long short-term memory cells for diacritizing the input Arabic poems.

A critical step in meter classification is the comparison with common patterns. Mojiri [23] and Yousefi [29] compare the poem with 31 common Persian patterns, Alabbas et al. [25] compare with 16 meters of Arabic, and Kurt & Kara [24] compares with 20 plain and 45 mixed Ottoman templates.

The efficiency of the rule-based works is varying. Mojiri [23] reports 65% precision, Jafarari Qamsari [27] accuracy of more than 98%, Alabbas et al. [25] precision higher than 96%.

The data-driven works have reported acceptable results. Yousefi [29] reports 92% of accuracy. Yousef et al. [28] report overall accuracy of 96.38%, Al-shaibani et al. [30] report more than 94% accuracy and Abandah et al. [31] report an average accuracy of 97.27%.

## 3 Kurdish poem meter classification

**Fig 1:**
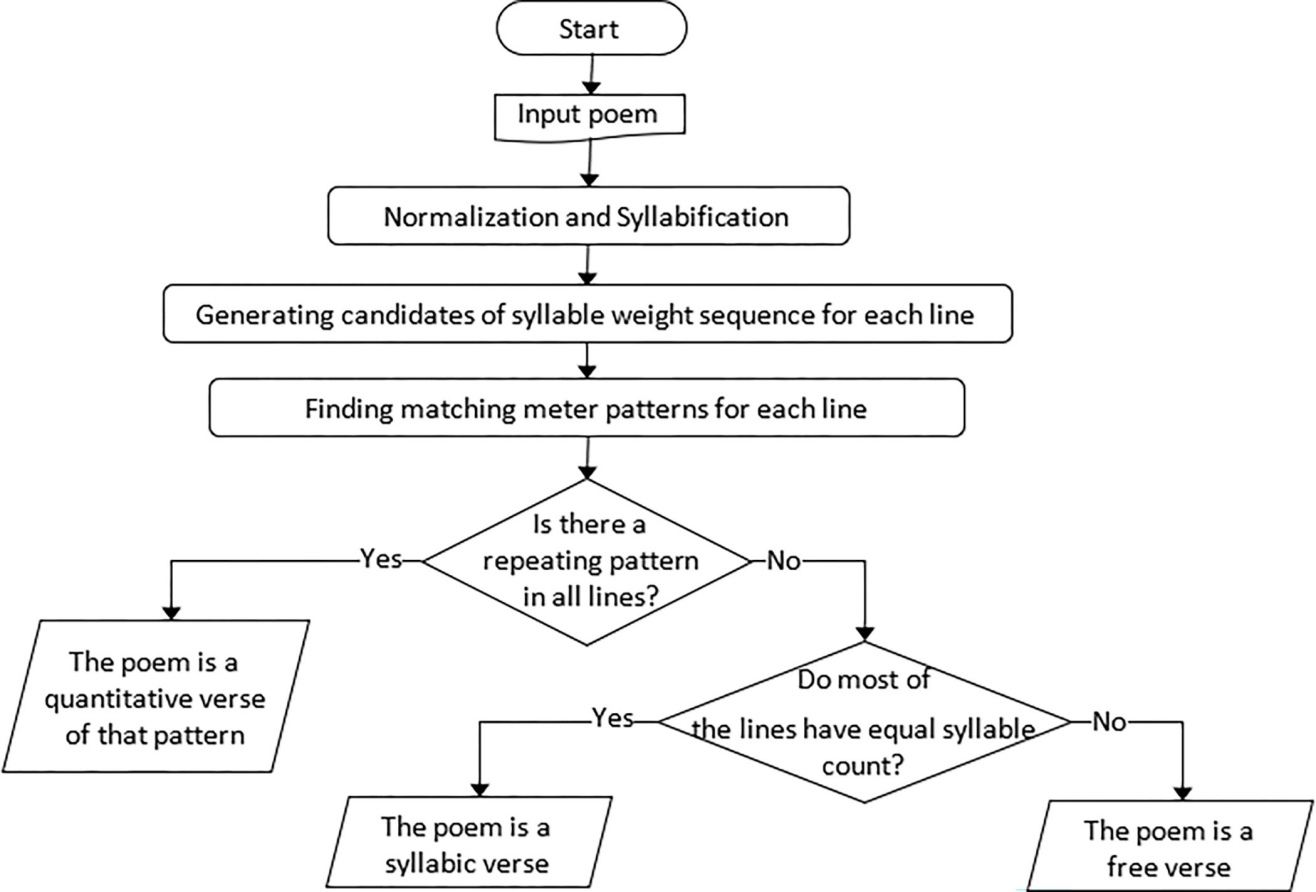
Flowchart of the proposed method.

In this section, we describe the proposed method in detail. The input is a Kurdish poem text written in the standard alphabet of Kurdish. The output is the type (quantitative, syllabic, or free verse) and the metrical pattern of the poem. The traditional manual method described earlier influenced our method of automatic meter classification. Fig 1 illustrates the flowchart of the proposed method. The method is available as a web application at https://asosoft.github.io/poem.

### 3.1 Normalization and syllabification

As the input of the proposed method is plain text, we perform the following normalization steps to prevent errors:

- Removing lines that contain plenty of non-Kurdish characters or have previously tagged as non-Kurdish.
- Converting some Arabic characters to the standard Kurdish equivalents, e.g., “ث” and “ص” into ‘س’.
- Converting punctuations and end-of-line characters to a plus mark (+) to demonstrate an “open juncture”.

Next is the syllabification process. For orthographies like English, Arabic, and Central Kurdish that do not have a one-to-one correspondence between the alphabet letters and the phonemes of the language, there will be challenges for syllabification. In this study, we use the rule-based method of SCK grapheme-to-phoneme conversion presented by Mahmudi & Veisi [5], which converts the input text into a syllabified string of phonemes. This method also correctly merges the conjunction ‘و’ (and) with the previous word [5]. This merge (e.g., “تیر و کەوان” */tî*.*rû ke*.*wan/* and “پۆڵا و ئاسن” */po*.*ław ʔa*.*sin/*) is required for the following scansion step.

For example, the following line by Nalî (1800–1877) will be normalized and syllabified as:

**Table:** 

the input:	گەر نەبەخشێ مەرهەمی وەصڵی، برینم کارییە
normalized:	گەر نەبەخشێ مەرهەمی وەسڵی+ برینم کارییە+
syllabified:	*ger ne.bex.şê mer.he.mî wes.łî* + *bi.rî.nim ka.rî.ye* +

Inside the input poem, some words may occur more than once, therefore, we store the syllabified sequences of phonemes for each word to speed up the process of syllabification.

### 3.2 Generating candidates of syllable weight sequence

As vowel length is not distinctive in Kurdish, for preserving the meter in quantitative verses, sometimes, short vowels should be pronounced long and long ones short. There are some clues for recognizing syllable weight changes automatically:

- **A:** Long vowels in word-final unstressed positions are pronounced short.
- **B:** When a short vowel precedes an open juncture (punctuations or the end of a line), it is usually pronounced long.
- **C:** In quantitative verses, that foot start with two adjacent light syllables, often, the first syllable is heavy, and for satisfying the meter, it should be pronounced light.
- **D:** When the long vowel */î/* in an open syllable precedes approximant */y/*, the vowel is pronounced short. E.g., */nî*.*ye/* ‘is not’.
- **E:** A syllable with two-consonant onset can also be pronounced as a two-syllable sequence (**˘ˉ**). For example, */xwa/* ‘god’ as */xu*.*wa/* and */gyan/* ‘soul’ as */gi*.*yan/*.

For managing the uncertainties in syllable weights, considering the above clues, we generate possible weight sequence candidates. For example, in */ger nebexşê merhemî wesłî birînim karîye/*, we have:

**Table:** 

	_1_	_2_	_3_	_4_	_5_	_6_	_7_	_8_	_9_	_10_	_11_	_12_	_13_	_14_	_15_
Syllable:	*ger*	*ne*	*bex*	*şê*	*mer*	*he*	*mî*	*wes*	*łî*	*bi*	*rî*	*nim*	*ka*	*rî*	*ye*
Normal weight:	ˉ	˘	ˉ	ˉ	ˉ	˘	ˉ	ˉ	ˉ	˘	ˉ	ˉ	ˉ	˘	˘
both weights are possible?	yes						yes		yes					yes	yes
meter pattern:	ˉ	˘	ˉ	ˉ	ˉ	˘	ˉ	ˉ	ˉ	˘	ˉ	ˉ	ˉ	˘	ˉ

- By clue C, syllable 1 can be pronounced light because we do not know the meter for now.
- By clue A, syllables 7 and 9 can be pronounced light.
- By clue D, syllable 14 is pronounced light.
- By clue B, syllable 15 can be pronounced heavy.

In the above example, for 5 syllables, there are 2 possible weights; therefore, 2^5 = 32 sequence candidates can be generated.

### 3.3 Finding the matching patterns for each line

As the quantitative meters have more detailed and are harder to compose, we first examine the lines of the poem for detecting a quantitative pattern. In this step, for each line, we compute the Levenshtein edit distance of each syllables weight sequence candidate with 27 common meter patterns (presented in Table 5). For example, if a line of the poem has 32 weight sequence candidates, we must calculate 32×27 = 864 edit distances. Since the strings are less than 20 characters long and contain only two characters (**˘** and **ˉ**), these calculations are done quickly.

For each line, we only store candidate-pattern pairs that have the smallest distances below a maximum acceptable distance (given 4). For example, for */ger nebexşê merhemî wesłî birînim karîye/*, among 864 pairs, only 62 pairs are acceptable, and one of these 62 pairs is:

- **ˉ˘ˉˉˉ˘ˉˉˉ˘ˉˉˉ˘˘** (syllable weight sequence candidate)
- **ˉ˘ˉˉˉ˘ˉˉˉ˘ˉˉˉ˘ˉ** (nearest common pattern, with an edit distance of 1)

### 3.4 Meter classification

The meter classification of a Kurdish poem, just by one or two lines is not correct at all the times, because:

- some lines of a syllabic verse may follow a quantitative pattern
- some lines may contain misspellings
- syllabification of some words and weight of some syllables are ambiguous
- unprofessional poets may commit mistakes in patterns

Therefore, in our proposed method, we consider all lines of the poem together. For each acceptable pair from the previous step, we add up to the score of the corresponding pattern for the whole poem. Eventually, there is a score for each common meter pattern. For example, if the pattern ˘ˉˉˉ/˘ˉˉˉ/˘ˉˉ has a small distance with most of the lines, its score will be higher. The pattern with the highest score is the most probable quantitative pattern of the poem; i.e., we define:

$$P=\underset{p_{j}\in M}{\mathrm{argmax}}\sum_{i=1}^{n}\left(MaxDist-Dist(p_{j},w_{i})\right)$$ (1)

In which, *P* is the most probable quantitative pattern of the poem, *M* is the set of common metrical patterns, *n* is the number of lines of the poem, *Dist(p*_*j*_,*w*_*i*_*)* is the edit distance of a pattern *(p*_*j*_*)* and weight sequence of a line *(w*_*i*_*)*, *MaxDist* is the maximum acceptable edit distances (given 4).

Sometimes the score of the winner pattern is close to another one and the victory is not decisive. Therefore, the most probable pattern should be regulated by a confidence criterion, according to the following formula:

$$Confidence=\frac{HighestScore}{MaxDist\times LinesCount}$$ (2)

The poem must have the following conditions to be recognized as a “quantitative verse”:

- Nearly all lines of the poem must have a same syllable count, i.e., the amount of standard deviation has to be small.
- The majority of lines must comply with a pattern, i.e., the calculated confidence has to be high.

If a poem fulfills only the first condition, the proposed method assigns it as a “syllabic verse” of the statistical mode of syllables count of lines. Else, if none of the above conditions are fulfilled with the poem, it will be classified as a “free verse”.

## 4 Results

### 4.1 Test dataset

We evaluated our proposed method on a dataset consisting of 1,154 Central Kurdish poems (979 quantitative, 130 syllabic, and 45 free verses) from available poems of “VejinBooks” (available at https://books.vejin.net). This website is a growing free online corpus of Kurdish literary texts in different dialects of Kurdish. The type and meter of all poems in this corpus are specified manually. VejinBooks also has statistics about the frequency of each meter available in the corpus. Among the available texts on the website, we chose only poems with more than three couplets from 12 well-known poets of Central Kurdish. Table 6 shows the overall statistics of the dataset. The dataset is available on GitHub at https://github.com/AsoSoft/Vejinbooks-Poem-Dataset (reference number 4079471).

**Table 6:** Statistics of poems of the dataset.

Poet	Quantitative	Syllabic	Free verse
Nalî _(1800–1877)_	125	-	-
Salim _(1800–1866)_	259	-	-
Kurdî _(1809–1850)_	77	-	-
Ḧacî Qadir _(1816–1897)_	102	-	-
Wefayî _(1844–1902)_	118	12	-
Ḧerîq _(1856–1909)_	49	1	-
Narî _(1874–1944)_	95	-	-
Qaniƹ _(1898–1965)_	56	13	-
Diɫdar _(1918–1948)_	18	11	-
Hêmin _(1921–1986)_	53	29	-
Herdî _(1922–2006)_	13	-	-
Kakey Felaḧ _(1928–1990)_	14	64	45
**Overall**	**979 (84.8%)**	**130 (11.3%)**	**45 (3.9%)**

The use of Arabic or Persian phrases (like Arabic Quranic Verses) within the text is a common convention in Kurdish poetry. Since our method is based on Central Kurdish phonology, this can be a problem for the evaluation. Fortunately, in the VejinBooks corpus, non-Kurdish phrases are tagged. We removed all the lines with a non-Kurdish phrase inside the test dataset.

### 4.2 Test results

We evaluated our method in type (quantitative, syllabic, or free) and pattern classification. The evaluation metrics are precision, recall, and F1-score. In Table 7, we show the results of the poem-type classification.

**Table 7:** Test Results for poem-type classification.

Poem Type	Count	Precision (%)	Recall (%)	F1-score (%)
Quantitative	979	99.4	97.4	98.4
Syllabic	130	83.2	95.4	88.9
Free verse	45	100.0	100.0	100.0
**Overall**	**1,154**	**97.3**	**97.3**	**97.3**

Table 8 indicates the test results for pattern classification. It shows the efficiency of the proposed method for each pattern. The recall for patterns that have “فعلاتن” (**˘˘ˉˉ**) or “فعلن” (**˘˘ˉ**) feet, like “فعلاتن مفاعلن فعلن”, is low. The method often classifies the poems of these patterns as syllabic. It causes a lower classification precision for the syllabic type, as shown in Table 7. Maybe the reason is that finding and matching words in the poem with two adjacent light syllables at the start of feet is hard in Kurdish. Therefore, poets consider using poetic licenses to preserve the meter.

**Table 8:** Meter pattern classification results, separated by pattern.

Meter Pattern	Count	Precision (%)	Recall (%)	F1-score (%)
مفاعیلن مفاعیلن مفاعیلن مفاعیلن	245	100	100	100
فاعلاتن فاعلاتن فاعلاتن فاعلن	224	99	100	99
مفاعیلن مفاعیلن فعولن	151	99	100	99
مفعولُ مفاعیلُ مفاعیلُ فعولن	117	99	99	99
فعلاتن فعلاتن فعلاتن فعلن	77	100	91	95
فاعلاتن فاعلاتن فاعلن	33	92	100	96
مفعولُ فاعلاتُ مفاعیلُ فاعلن	31	100	100	100
فعلاتن مفاعلن فعلن	25	100	20	33
مفعولُ مفاعیلن مفعولُ مفاعیلن	16	100	100	100
فعلاتن فعلاتن فعلن	13	100	77	87
مفعولُ فاعلاتن مفعولُ فاعلاتن	10	90	90	90
مفاعلن فعلاتن مفاعلن فعلن	7	100	57	73
مفعولُ مفاعلن فعولن	5	100	100	100
مفتعلن مفاعلن مفتعلن مفاعلن	4	100	75	86
مفتعلن فاعلن مفتعلن فاعلن	4	80	100	89
مستفعلن مستفعلن مستفعلن مستفعلن	4	80	100	89
فعولن فعولن فعولن فعل	3	75	100	86
فاعلاتن فاعلاتن فاعلاتن فاعلاتن	3	100	100	100
مفتعلن مفتعلن فاعلن	2	100	100	100
فعلاتُ فاعلاتن فعلاتُ فاعلاتن	2	100	50	67
مفعولُ مفاعیلُ مفاعیلُ فعل	1	100	100	100
مفاعیلُ مفاعیلُ مفاعیلُ فعولن	1	0	0	0
مفاعلن فعولن مفاعلن فعولن	1	25	100	40
مفاعلن مفاعلن مفاعلن مفاعلن	0	0	0	0
مفاعلن فعلاتن مفاعلن فعلاتن	0	0	0	0
16-syllabic	9	100	89	94
15-syllabic	6	100	100	100
14-syllabic	8	55	75	63
13-syllabic	4	100	100	100
12-syllabic	5	100	100	100
11-syllabic	8	100	63	77
10-syllabic	49	71	100	83
8-syllabic	31	100	100	100
7-syllabic	10	100	100	100
free verse	45	100	100	100
**Overall**		**96.2**	**96.2**	**96.2**

**Fig 2:**
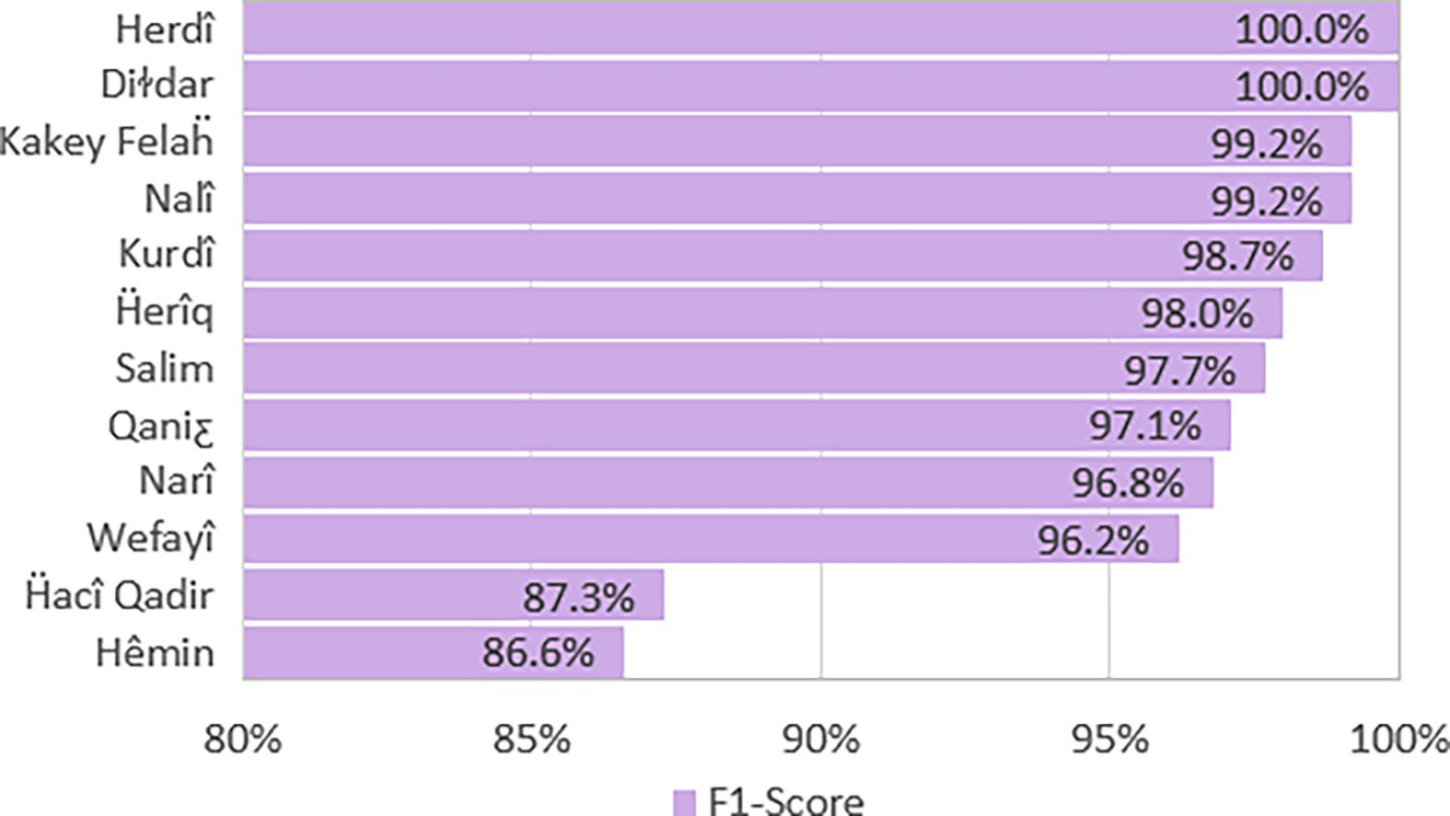
Metrical pattern classification results, separated by poet.

Fig 2 shows the test results for metrical pattern classification, separated by authors. It can be speculated how much a poet complies with the patterns and uses fewer poetic licenses. For example, Herdî is known for having few but admirable poems. The lower accuracy for the poems of Hêmin and Ḧacî Qadir is due to using patterns with “فعلاتن” (**˘˘ˉˉ**) or “فعلن” (**˘˘ˉ**) feet and using more poetic licenses.

## 5 Conclusions and future works

In this paper, we have proposed an automatic poem meter classifier for the Central Kurdish language. The evaluations achieved an overall precision of 97.3% in meter-type classification and overall precision of 96.2% in metrical pattern identification.

In the future, we plan to extend the method’s functionality in identifying subclasses of Kurdish free verses. Automatic author identification, based on the poem’s characteristics, is another field of study for further works. Since Kurdish is a low-resourced language, our rule-based classifier can help with the tedious task of data preparation for future machine-learning solutions.

Now, this algorithm assists the contributors of Vejinbooks online corpus in tagging newly imported poems. Furthermore, an online application is developed for amateur poets to evaluate their poems.
